# Acrobustitis-phimosis in bulls: postoplasty technique performed with the animals in a standing position

**DOI:** 10.1590/1984-3143-AR2023-0047

**Published:** 2023-09-18

**Authors:** Monike Alves Lopes, Frederico Ozanam Papa

**Affiliations:** 1 Universidade Estadual Paulista, Faculdade de Medicina Veterinária e Zootecnia, Botucatu, SP, Brasil

**Keywords:** acroposthitis, acrobustitis surgery, impotentia coeundi

## Abstract

Acrobustitis is the inflammation of the distal prepuce, which can lead to a narrowing of the preputial ostium due to stenosis or growth of fibrous tissue after an inflammatory reaction. This condition usually occurs in cattle with long prepuce, such as Zebu or Zebu’s crossbreeds, leading the animal to Impotentia Coeundi, this condition is characterized by the bull's disability to copulate, that leads to lower herd fertility and consequent financial losses. Normally, corrective surgeries are performed on-farm and the animal is placed in a lateral recumbency. However, in some situations the animal is restrained with ropes and remains on the grass, dirt or even on uneven floors, which can cause neuropathies, bloat or hypoxia. Due to a series of complications that can occur in the postoperative period of surgery in the lateral recumbency, this article aims to describe the surgical technique for correcting acrobustitis with the animal in a standing position. Ten corrective surgeries for acrobustitis were performed in adult bulls between 4 and 8 years of age and predominantly of zebu or crossbreeds, with a total recovery of the animals for full reproductive activity.

## Introduction

**Figure 1:**
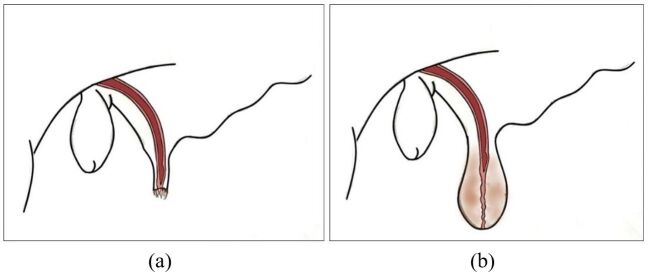
Graphic representation of normal prepuce (a) and a prepuce with acrobustitis (b). Illustration: Luis Fernando Mercês Chaves Silva.

Acrobustitis is the inflammation of the extremity of the prepuce (Figure 1), which usually leads to a narrowing of the preputial ostium (phimosis), causing difficulty or the impossibility of the bull to expose the penis (Bello, 2020). This condition is classified as one of the causes of impotentia coeundi, which is the type of impotence related to the inability of a male to copulate (Rabelo and Silva, 2011). The narrowing of the preputial ostium or its partial obstruction makes it impossible for the breeder to perform natural mating or semen collection by artificial vagina (Nascimento and Santos, 2021).

Acrobustitis frequently affects cattle with long prepuce, such as zebu cattle (Carvalho, 2020). Due to the predominance of the Nelore breed in Brazil, this condition is relevant in the country, since it can lead to low fertility rates and consequent financial losses. Other possible causes of acrobustitis include disorders of the retractor muscle of the penis, failure to control ectoparasites, management failure, pasture with thorny plants, grasses such as Colonião (Panicum maximum), Napier (Pennisetum purpureum) and others that have serrated and sharp edges, deforested pastures with remains of trees or weeds that can cause trauma to the exposed prepuce, or even bird pecks and accidents with barbed wire and other sharp objects (Riet-Correa et al., 2011).

The inflammation initially causes edema, that can lead to prolapsing of the inner layer of the prepuce, which brings it even closer to the ground, causing further damage if the animal is not treated quickly and removed from the site where the injuries occurred. If not treated, the tissue grows and the injury intensifies with more lesions and ulcerations (Sousa et al., 2018; Silva et al., 2019).

Most of the acrobustitis surgeries are performed on-farm, usually with the bulls in lateral recumbency (Morin et al., 2002), and this conduct may bring some postoperative complications. Important complications may occur, such as lesions of the radial nerve (Pereira et al., 2008), bloat, regurgitation, and brinchoaspiration (Hendrickson and Baird, 2013). These techniques can be performed with the animal in two different restraining positions: lateral recumbency and in standing position. In this study, the efficacy of the postoplasty in standing position was evaluated in 10 different cases.

## Methods

**Figure 2:**
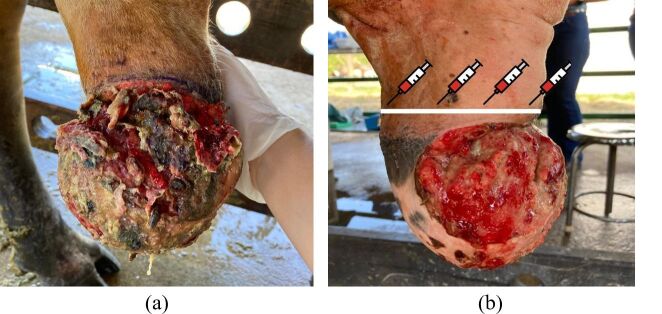
Bull preputial aspect on day one after it arrived at the veterinary hospital (a), the aspect of the prepuce after the initial treatment, and a scheme for local application of 2% Lidocaine, around the preputial perimeter where the incision will be performed (b).

From 2012 to 2022, 10 zebu or crossbred bulls, between 4 and 8 years old, with swelling and ulceration in the preputial extremity (Figure 2a) were treated. Upon clinical examination, the animals presented vital parameters considered normal for the species. The development of this research was approved by the Animal Research Ethics Committee of the São Paulo State University (UNESP - Botucatu) (approval number: 0280/2022).

In order to reduce inflammation, initial treatment was performed with local shower with cold water and extensive cleaning of the wound twice a day for five days, the application of non-steroidal anti-inflammatory drug, flunixin meglumine, at a dose of 1.1 mg/kg once a day for 3 days, and the surgeries were performed five to seven days after this initial treatment (Figure 2b).

**Figure 3:**
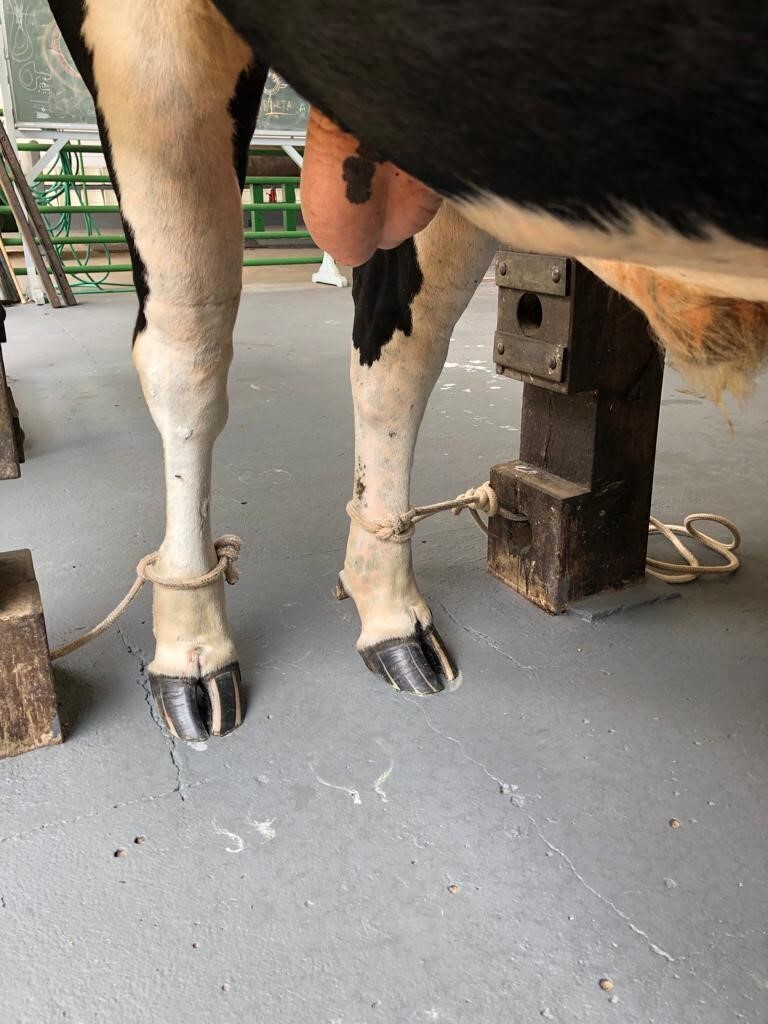
Restraining the bull to perform the procedure.

The animals were fasted for 8 hours before the procedure and placed in a restraint trunk with ropes tied around the pastern to avoid kicking (Figure 3). Sedation was administered with intramuscular injection of 2% Xylazine, at a dose of 0.2 mg/kg (Greene, 2003; Mulon, 2019). For local anesthetic blockade (Skarda, 1996; Nagy, 2019), 2% Lidocaine was injected on the incision line across the preputial circle between the intact and injured regions (Figure 2b). After performing the local blockade, still with the animal in the standing position, the skin incision commenced.

The skin incision was made with a scalpel around the preputial perimeter, above the injured site. After skin incision, divulsion of the subcutaneous tissue was performed with Metzembaum scissors, until reaching the preputial mucosa. Hemostasis of small vessels was made with hemostatic forceps, or ligature with absorbable surgical thread Polyglactin 910 2-0, for larger vessels.

**Figure 4:**
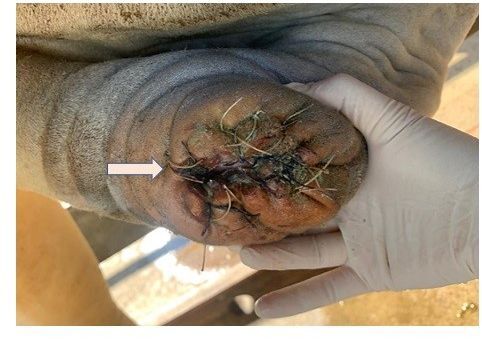
Suture of the preputial mucosa to the skin with Wolff Stitches and the arrow showing the “V” incision in the caudal position of the preputial ostium.

After the removal of the affected tissue, four Allis or Kocher forceps were used to pinch the preputial mucosa at the four cardinal points so that the anatomical position of the prepuce was maintained until the end of the procedure. Afterward, an incision was made in the preputial mucosa, caudal position, forming a “V” in the new preputial ostium, to prevent cicatricial retraction and ostial stenosis (Figure 4).

The suture of the preputial mucosa to the skin was started initially at each cardinal point where it was pinched and following the sutures in the rest of the preputial perimeter, with non-absorbable nylon 0 thread or Braunamid White USP 3, Metric 6 de 50m (B/BRAUN, Aesculap AG&CO KG, D-78532 Tuttligen-Germany). Wolff stitches were performed, starting on the external skin tissue, internal skin tissue, internal preputial mucosa, returning through the internal and external preputial mucosa, internal and external skin and the tying of the stitches on the external skin tissue, preventing it from being on the inner side, which would impair wound healing (Figure 4).

**Figure 5:**
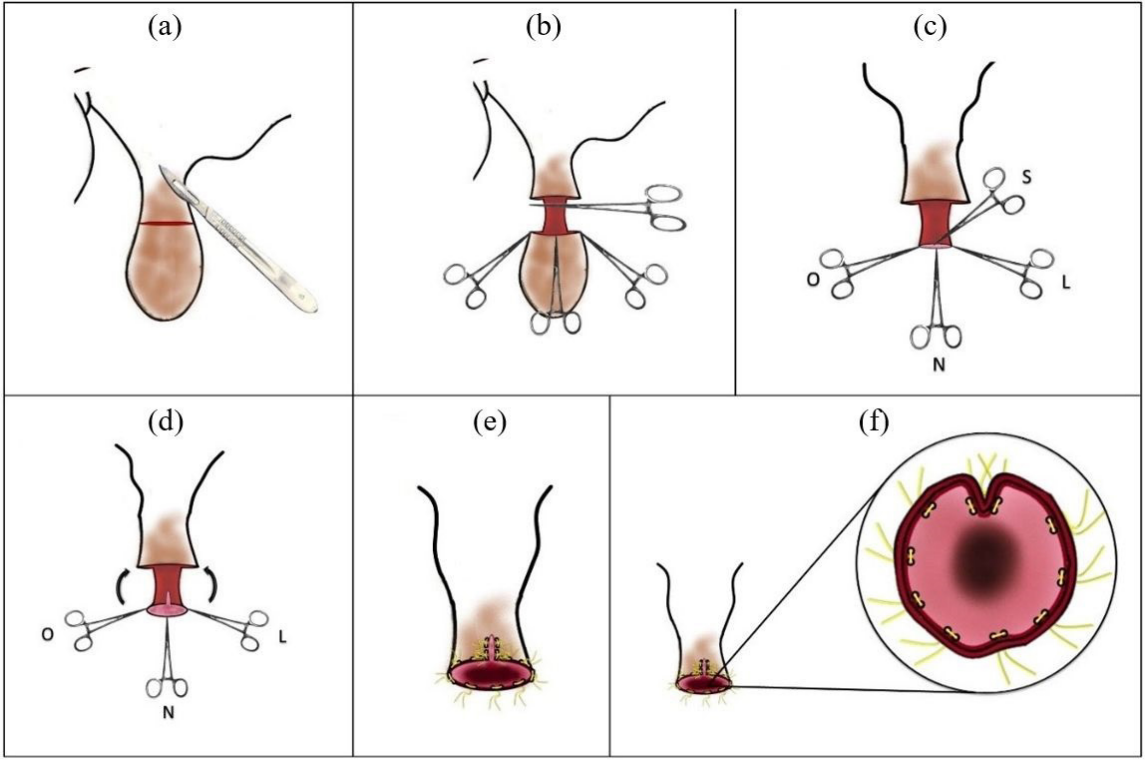
Graphical representation of the stages of the postoplasty surgery. Lateral view of the skin incision (a), divulsion of subcutaneous tissue and exposure of the inner lamina of the prepuce and demarcation of the cardinal points with forceps (b), excision of the affected area (c), caudal view of the incision in the caudal region of the preputial mucosa and positioning (arrows) to start the suture (d), interrupted Wolff suture (e) and ventral view of the surgical wound (f). Illustration: Luis Fernando Mercês Chaves Silva.

Immediately after surgery, the antibiotic penicillin benzathine was applied at a dose of 30,000 IU/kg, being reapplied after 48 hours (totalling 2 applications). Additionally, a non-steroidal anti-inflammatory drug, flunixin meglumine, was applied at a dose of 1.1 mg/kg, once a day, for 3 days, both intramuscular. Wound dressings were made twice a day, consisting of cleaning with saline solution and gauze, application of chlorhexidine-based ointment, repellent powder, and silver sulfadiazine larvicide spray around the prepuce to prevent myiasis. Note the graphic representation of the stages of the postoplasty surgery below (Figure 5).

## Results

A few days after surgery the bulls started gradually exposing the penis normally at the time of urination. On average, the healing of surgical wound and removal of the stiches occurred after 7 days post-surgery. Only three animals had postoperative complications (suture dehiscence) and the therapeutic conduct was appropriate for the situation, extending the use of antibiotic to 1 more application (total 3 applications) and wound dressings until complete healing, which occurred on average 10 to 15 days after surgery. After removing the stitches and total healing of the surgical wound, all animals had semen collected with an electroejaculator (all of them exposed the penis normally). All bulls were considered capable of reproductive activity, and they returned to their properties. The animals remained in the field in a natural mating regime, and according to information from the owners, the bulls returned to reproductive activity with fertility rates within normal limits.

## Discussion

Due to it being naturally heavy animals, the prolonged decubitus, often associated with not using padded material during surgery performed in lateral recumbency, can promote complications in cattle, such as radial nerve injury (Pereira et al., 2008), as described by Jurado et al. (2011).

Prolonged lateral recumbency can also cause bloat, regurgitation, and hypoxia. As a consequence of being in unnatural position for the cattle, the ruminal fluid blocks the cardia, preventing proper eructation and increasing the intraruminal gas (HuiChu, 2022). The pressure to the diaphragm makes breathing difficult and causes hypoxia and acidosis (Walker and Vaughan, 1980; Carroll and Hartsfield, 1996; Seddighi and Doherty, 2016).

Hypoxia and anoxia may also occur, in part due to bloat and regurgitation, but other factors are also involved, such as respiratory distress due to compression of the lower lung lobe (Abrahamsen, 2008). The anesthetic depression can make breathing more diaphragmatic, but the abdominal organs put pressure on it, making it unable to adequately perform its function, then causing hypoxia or anoxia, which leads to acidosis (Walker and Vaughan, 1980; Carroll and Hartsfield, 1996; Seddighi and Doherty, 2016).

Likewise, osteoarticular injuries can also occur due to the use of ropes on the animal's limbs, both at the time of the overthrow and in the restrain itself. These conditions range from fractures to luxation (Blowey, 2016).

In a study with orchiectomy in bovines in the standing or lateral recumbency position it was observed that the lateral recumbency made it difficult for the surgeon to perform the technique, in addition to requiring trained personnel with greater physical strength to imobilize and restrain the animal (Silva et al., 2003). This information corroborates with the proposal of this article, that the standing surgical technique may require fewer people and physical strength for restraint the bull, as well as providing better positioning of the surgeon during the procedure.

## Conclusion

The postoplasty with the bull in a standing position is an efficient surgical technique and is advantageous in many ways, both regarding to animal welfare by preventing injuries, which consequently prevent financial losses to the property, as well as providing better positioning and comfort for the surgeon during the procedure.
